# New nano-ferro-silicon biochar promotes plant growth and grain yield under arsenic stress in rice

**DOI:** 10.3389/fpls.2025.1556696

**Published:** 2025-05-02

**Authors:** Chaowei Fang, Boran Dong, Shengyue Ye, Lei Zhang, Qingpo Liu

**Affiliations:** ^1^ College of Advanced Agricultural Sciences, Zhejiang A&F University, Hangzhou, China; ^2^ The Agricultural Industrialization Development Service Center of Tonglu County, Hangzhou, China

**Keywords:** rice, new nano-ferro-silicon biochar, arsenic stress alleviation, plant growth promotion, grain yield

## Abstract

Arsenic (As) is a ubiquitous and toxic metalloid in nature, posing significant risks to living organisms. Developing sustainable strategies to mitigate As stress and reduce As accumulation in rice is critical for ensuring food safety in contaminated regions. Herein, we synthesized a new nano-ferro-silicon biochar (NNFB) composed of biochar, γ-Fe_2_O_3_, and SiO_2_, which effectively adsorbed As from aqueous solutions and soil. NNFB alleviated As toxicity by promoting rice seeding and root growth at the seed germination and seeding stages. Under 40 μM As(III) treatment, application of 0.25% and 0.5% NNFB regulated the reactive oxygen species (ROS) balance by reducing H_2_O_2_ accumulation and enhancing peroxidase (POD) activity in leaves. Additionally, NNFB reduced As uptake by regulating the expression of As transport genes *OsABCC1*, *OsLsi1*, and *OsLsi2* at the seeding stage. In pot experiments with 40 mg/kg As(III)-contaminated soil, NNFB application significantly improved aboveground biomass, tiller number, and effective tiller count. Notably, seed number per plant increased by 6.93- and 7.93-fold in 0.5% and 1% NNFB treatments compared to the control. These findings demonstrate that NNFB efficiently adsorbs As, mitigates As stress at multiple growth stages, and enhances rice productivity, offering a promising solution for As-contaminated agricultural systems.

## 1 Introduction

Arsenic (As) is widely distributed in nature and commonly associated with various metal ores, including copper, lead, and gold, which is found in both inorganic and organic forms (Oremland and Stolz, 2003). The inorganic forms of As, commonly observed as oxides, sulfides, arsenate (AsV), and arsenite (AsIII), are readily absorbed by plant roots (Cullen and Reimer, 1989; Drobna et al., 2009; Finnegan and Chen, 2012). The contamination of water and soil by elevated concentrations of As has been documented in multiple regions globally, necessitating urgent attention towards the effective remediation and control of global As pollution (Mohanty, 2017).

As could enter the food chain through the consumption of As-contaminated rice, which is a staple food of the world (Meharg et al., 2009). Plant growth and development could be significantly impacted by As exposure. When cultivated in As-contaminated soil and water, plants may accumulate substantial amounts of As in their kernels or edible shoots, thereby entering the food chain (Zhang et al., 2021). As absorbed in disparate forms because of its different forms in plant root. The AsV was absorbed under aerobic condition; however, AsIII was the main form in anaerobic condition, such as flooded paddocks and rice fields (Clemens and Ma, 2016; Huang et al., 2020; Lindsay and Maathuis, 2017). In plant cell, the translocation of As is mainly through two major pathways: extruded out of the root cells via bidirectional aquaporin channels and detoxified by compartmentalization into vacuoles followed by its complexing with thiol compounds (Zhao et al., 2010; Pickering et al., 2000; Raab et al., 2005; Song et al., 2014). Excess of As not only broke the balance of reactive oxygen species (ROS), resulting in lipid peroxidation and damage to cellular membranes in plant (Tripathi et al., 2015), but also impacted on many metabolic processes, including glutathione metabolism (Foyer and Noctor, 2011), photosynthesis (Chandrakar et al., 2016; Siddiqui et al., 2020), carbohydrate metabolism (Jha and Dubey, 2004), lipid metabolism (Duwiejuah et al., 2020; Singh et al., 2009; Srivastava et al., 2005), protein metabolism (Ismail, 2012; Singh et al., 2009, 2006), and sulfur metabolism (Huang et al., 2020; Benito et al., 2014).

Biochar, produced through the pyrolysis of carbon-rich solid biomass under oxygen-limited conditions, enhances plant nutrient uptake by supplying essential nutrients to plants and soil microorganisms (Haider et al., 2022; Lusiba et al., 2017). The raw materials for biochar production could be sourced from agricultural residues (e.g., crop straws and animal manures) and municipal solid waste (e.g., food wastes, sewage sludge, and woody residues). Additionally, other potential sources such as shells and chitin could also be utilized (Duwiejuah et al., 2020). Biochar enhanced the soil properties, such as aggregate stability, porosity, soil bulk density, water holding capacity, and nutrient retention due to its substantial surface area (Lehmann et al., 2006). Moreover, biochar facilitated the assimilation of essential nutrients, such as phosphorus (P), potassium (K), calcium (Ca), magnesium (Mg), sodium (Na), aluminum (Al), iron (Fe), copper (Cu), and zinc (Zn) in both the above- and belowground growth of plants (Sigua et al., 2016). The application of biochar also reduced heavy metals accumulation in plants (Puga et al., 2015; Houben et al., 2013; Yu et al., 2017; Wang et al., 2020). Application of biochar resulted in a decrease in As concentration across different parts of rice, which can be attributed to the oxidation process converting AsIII into AsV (Yu et al., 2017). In a word, the diversification of biochar holds significant potential to increase agricultural productivity, particularly in terms of soil amelioration.

The emergence of nanomaterials, a new material endowed with exceptional properties unattainable by conventional counterparts, had been used in many fields. In plants, nanomaterials facilitated growth, development, and improved stress resistance, while they also exhibited toxicity within a specific concentration range (Verma et al., 2018; Rizwan et al., 2019). Silicon (Si) could not only promote the growth and increase grain yield but also enhance the ability to resist various stresses of rice (Detmann et al., 2012). Applicating the exogenous silicon or silicon fertilizer alleviated the growth inhibition of seed germination and seedlings in rice under arsenic stress and reduced the accumulation of As in stems, leaves, and seeds of rice (Guo et al., 2005; Bogdan and Schenk, 2008; Liu et al., 2014). Fe, one of the essential elements of plants, played vital roles in plant physiological activities such as photosynthesis, respiration, the scavenging of reactive oxygen species, the alleviating As stress, and many other biological processes (Murgia et al., 2022; Wen et al., 2021). However, the biochar containing nanomaterials, Si and Fe elements for adsorbing As in the environment, and the mechanism of its alleviation of As stress at all growth and development stages in rice should be further investigated.

In this study, we provided a new nano-ferro-silicon biochar (NNFB), which contained nanomaterials, Si and Fe elements, synthesized through the pyrolysis method. The surface properties of the biochar were characterized by scanning electron microscopy (SEM), X-ray diffraction (XRD), and X-ray photoelectron spectroscopy (XPS). Then, we tested the adsorption of NNFB to As and its alleviation effect on As pollution at germination, seeding, and maturity stages of rice.

## 2 Materials and methods

### 2.1 Method for preparation of new nano-ferro-silicon biochar

**Figure 1:**
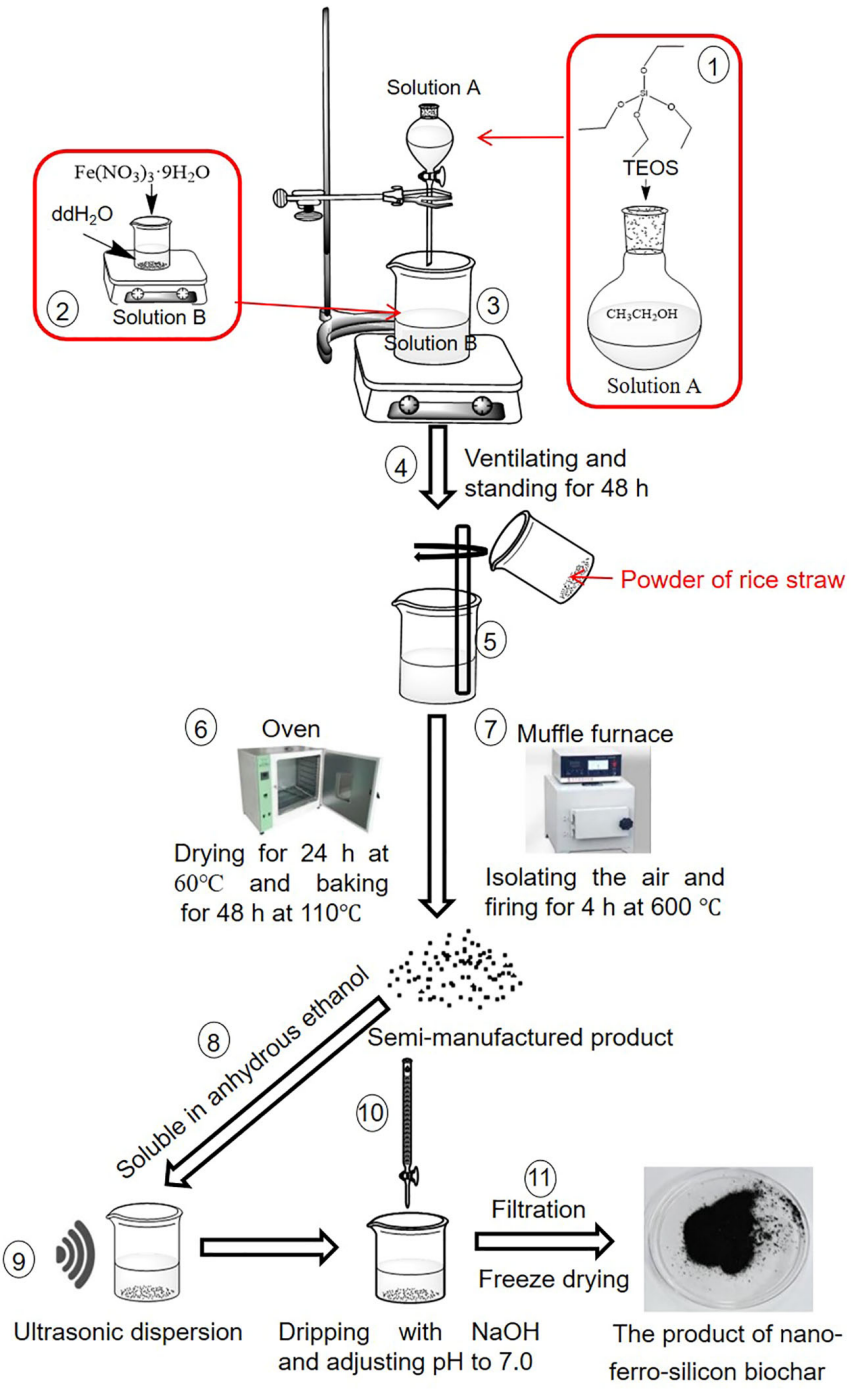
The manufacturing process of new nano-ferro-silicon biochar (NNFB).

For the NNFB production, step 1, the 11.2 mL tetraacetoxysilane (TEOS) and 3.79 g Fe(NO_3_)_3_·9H_2_O were dissolved into 8 mL anhydrous ethanol and 8 mL deionized water, respectively, to obtain solution A and solution B. For step 2, solution A was added to solution B slowly while stirring constantly. For step 3, the solution was ventilated at room temperature for 48 h statically to obtain a reddish-brown colloid. For step 4, the reddish-brown colloid was mixed with 6 g of rice straw powder thoroughly and evenly; then, the mixture was dried in an oven at 60°C for 24 h and at 110°C for 48 h in turn to obtain a tan powder, which was named sample C. For step 5, the sample C was wrapped in tinfoil tightly, then it was put into a muffle furnace and fired at 600°C without air for 4 h. For step 6, the powder obtained from step 5 was dissolved into anhydrous ethanol, then dispersed it under ultrasound for 15 min so that the component was distributed evenly. For step 7, the ethanol was filtered, and all the solid matter was collected. For step 8, the solid matter collected in the previous step was dissolved in 30 mL deionized water and dispersed evenly. For step 9, the pH was adjusted to 7.0 by NaOH. For step 10, the solid–liquid mixture was filtered using ultrafiltration membrane. For step 11, the solid matter was collected and then placed it into a freeze–drying machine at −50°C and 5 Pa for 24 h to obtain the final product, the new nano-ferro-silicon biochar (NNFB) (**Figure 1**).

### 2.2 Analysis of X-ray powder diffraction

The X-ray powder diffraction (XRD) detection was conducted using an X-ray diffractometer (Bruker, Germany). The details were performed according to the method described previously (Wen et al., 2021).

### 2.3 Analysis of X-ray photoelectron spectrometer and scanning electron microscopy

The X-ray photoelectron spectrometer (XPS) was tested by X-ray photoelectron spectroscopy (Shimadzu, Japan). The spot size and energy step were 400 μm and 1.0 eV, respectively. The XPS map was analyzed using Avantage software. The details were performed according to the method described previously (Wang et al., 2021). The structure and surface morphology of the samples were characterized using SEM (Zeiss, Germany).

### 2.4 Plant material and As treatments

The *Oryza sativa* L. *indica* rice cultivar 93-11, which is sensitive to As stress (Hu et al., 2013), was selected as experimental material. Rice seeds were disinfected with 75% ethanol and 0.5% NaClO and then subjected to accelerate germination at 37°C. The germinated seeds were carefully selected using tweezers and evenly distributed onto a black foam float plate that was covered with fine black gauze mesh (with an aperture size of 1 mm) and then placed in 2 L of rice nutrient solution (the formula of rice nutrient solution is shown in **Supplementary Table S1**). The rice growth condition was follows: light intensity of 6,000 lx, 16 h of light and 8 h of darkness per day at maintained temperature of 28°C, and humidity of 60%. To determine the effect of NNFB on the mitigating As stress, the experiment on treatment of rice seedlings cultured for 21 days in the above experiments by 40 μM NaAsO_2_ solution and NNFB was carried out. **Supplementary Table S2** shows the operations for each group. Each treatment group was set up with three replicates and growth for 3 days in the following conditions: constant temperature at 28°C, light of 18,000 lx, light for 16 h and darkness for 8 h in 1 day, and humidity of 60%.

The rice root length and bud length were also determined using the 7-d seeding under treatment with 0 μM, 20 μM, 40 μM, and 60 μM As(III) or treated with 40 μM As(III) with either 0.15% NNFB, NNFB A, and NNFB B.

### 2.5 Soil preparation for pot experiment

The tested soil contained 40 mg/kg As(III) along with three different concentrations of NNFBs (0%, 0.5% and 1% of mass ratio), which were mixed well within the tested soil before the rice planting. The CO(NH_2_)_2_ and KH_2_PO_4_ were applied to the collected soil and incubated under flooded conditions for 14 days for subsequent experiments. The contents of nitrogen, phosphorus, and potassium were 128, 89, and 71 mg/kg in soil, respectively. The crops were planted in mid-April and harvested in mid-September in Lin’an District, Hangzhou City, Zhejiang province.

### 2.6 Analysis of diaminobenzidine staining

As described previously (Guo et al., 2024), approximately 1 cm of seedling leaves were clipped and immediately placed into a centrifugation tube containing 5 mL of diaminobenzidine (DAB) dye solution and stained at room temperature until an obvious phenotype appeared (approximately 14 h). After discarding the dye, the leaves were left in the tube, 5 mL of 95% ethanol was added, and decolorization was performed in a water bath at 80°C, changing the same amount of ethanol every 10 min until the leaves are translucent white. The staining was observed under a stereomicroscope.

### 2.7 Analysis of peroxidase and catalase activity

The leaves of 21-d seedings treated with 40 μM As(III), 40 μM As(III) + 0.25% NNFB, and 40 μM As(III) + 0.5% NNFB for 7 days were prepared to test peroxidase (POD) and catalase (CAT) activities by spectrophotometry according to Pawłowska et al. (2021) and Liu et al. (2015). The rates of oxidation in the presence of H_2_O_2_ within 30 s were determined. CAT activity is expressed as U mg^−1^ protein min^−1^.

### 2.8 Analysis of total arsenic concentration

Totally, 0.2–1.0 g of dried As-treated 21-d seedlings was digested with 5 mL of HNO_3_ overnight in a digestion tank, which was then placed in a constant temperature drying oven at 80°C for 1–2 h, followed by 120°C for 1–2 h, and finally at 160°C for 4 h. After cooling naturally, deionized water was added to the digestion solution to a final volume of 25 mL. The total As concentration was quantified by inductively coupled plasma mass spectrometry (ICP-MS, Thermo Fisher RQ, USA), as described previously (Huang et al., 2024; Sun et al., 2024).

### 2.9 RNA extraction and quantitative real-time PCR

Total RNA was extracted from different tissues using TRIzol reagent (Yeasen, Shanghai, China). After treatment with DNase I, the reverse transcription reaction was performed according to the protocol of the RT system (Yeasen, Shanghai, China). Quantitative real-time PCR (qRT-PCR) was conducted in a Bio-Rad CFX96 Real-Time PCR system (Bio-Rad, USA) using the Hieff qPCR SYBR Green Master Mix (No Rox) (Yeasen, Shanghai, China). The reaction procedure for qRT-PCR was as follows: 95°C for 5 min, followed by 40 cycles of 95°C for 15 s, 60°C for 30 s, and 72°C for 30 s. The total volume of the reaction system was 20 μL, including Hieff^®^ qPCR SYBR Green Master Mix, 10 μL; forward primer, 0.4 μL; reverse primer, 0.4 μL; template of cDNA, 9 μL; and RNase-free water, 0.2 μL. The *Ubquitin* gene was used as the internal control. The transcription levels were calculated by the 2^−△△CT^ values. The reaction was performed as described previously (Zhao et al., 2023; Li et al., 2024). The primers used in this experiment are listed in **Supplementary Table S3**. The single sample had three biological replicates and three technical replicates.

### 2.10 Statistical analysis

The significance of treatment effects was assessed by variance analysis using the GraphPad Prism (v10.0) software. The ANOVA and Tukey’s test were conducted to evaluate the statistical difference between treatments or samples, where p-value <0.05 was considered as significantly different.

## 3 Results

### 3.1 Physicochemical properties of NNFB

**Figure 2:**
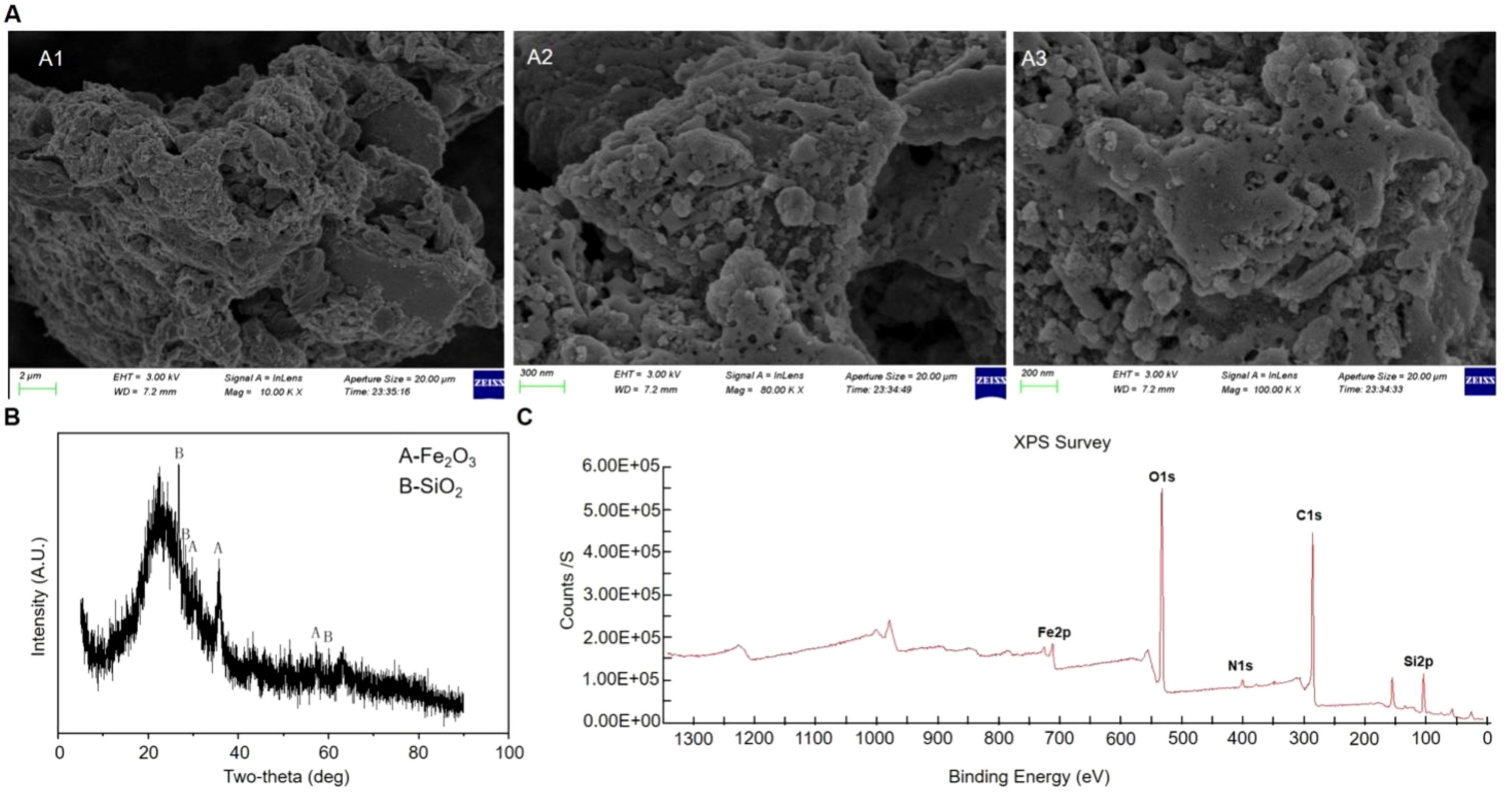
Characterization analysis of NNFB. **(A)** SEM analysis of NNFB. Parts (A1–A3) were obtained at 10K X, 80K X, and 100K X, respectively. **(B, C)** XRD and XPS analysis of NNFB, respectively.

The morphology of NNFB was characterized by SEM analysis, which revealed a block-like structure with distinct pore formations on the irregular surface (**Figure 2A**). This distinctive feature significantly increased the specific surface area of NNFB, enabling it to not only support nanomaterials efficiently but also exhibit excellent adsorption capacity.

The XRD was used to scan the range of 2θ = 0°–100° (**Figure 2**). All samples disclosed dispersion peaks at 30.283°, 35.677°, and 57.259° (2θ), and 26.827°, 29.695°, and 60.508° (2θ), which were identified as the characteristic peaks of γ-Fe_2_O_3_ and SiO_2_ crystals, respectively (**Figure 2B**), suggesting that the surface of NNFB was decorated with γ-Fe_2_O_3_ and SiO_2_ crystals. Moreover, the NNFB demonstrated distinct magnetic properties and was successfully attracted by an external magnet, aligning with the XRD analysis presented earlier.

To further understand the elemental composition and related structural information of NNFB surface, X-ray photoelectron spectroscopy (XPS) was used to detect NNFB. The results exhibited the characteristic peaks for Fe2p (700–740 eV), O1s (528–536 eV), N1s (380–420 eV), C1s (282–290 eV), and Si2p (100–108 eV) (**Figure 2C**). Taken together, the evident pore was observed on the surface of NNFB, with γ-Fe_2_O_3_ and SiO_2_ attached, and incorporating various elements within the NNFB.

### 3.2 NNFB is adsorbent for As

**Figure 3:**
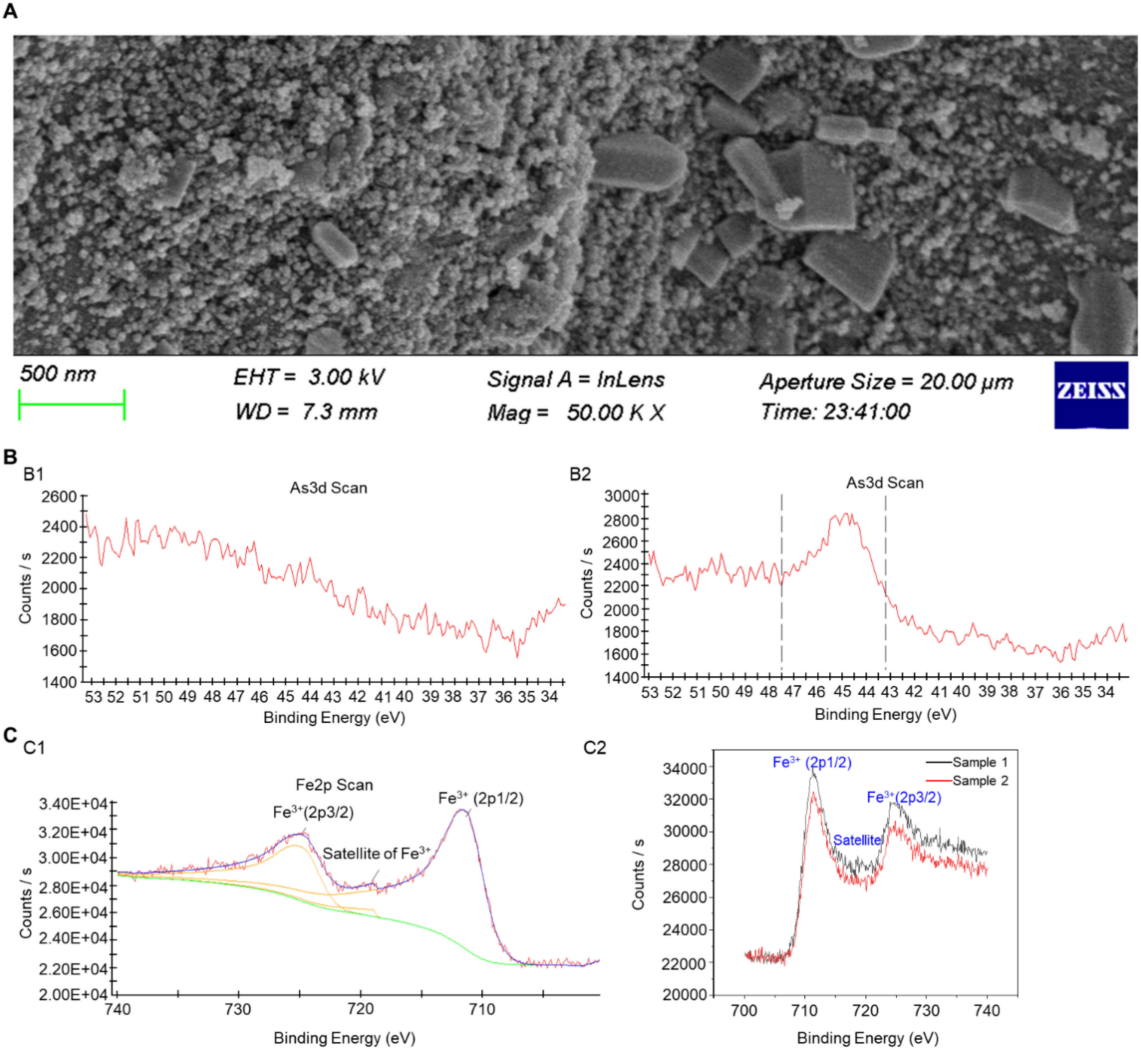
Characterization analysis of NNFB after full adsorption in As(III). **(A)** SEM analysis of NNFB after full adsorption in As(III). **(B)** As3d analysis of NNFB (B1) and NNFB after full adsorption in As(III) (B2), respectively. **(C)** Fe_2_P analysis of NNFB (C1) and NNFB after full adsorption in As(III) (C2), respectively.

The original NNFB and the NNFB after complete adsorption in the NaAsO_2_ solution were designated as sample 1 and sample 2, respectively. Hereinafter, the NaAsO_2_ solution was named as As(III). SEM analysis revealed a significant reduction in the pore structure on the surface of sample 2 compared to sample 1 (**Figures 2A**, **3A**), indicating that a large number of particles were attached to the surface of the NNFB. Furthermore, the As3d fine spectra confirmed that there was no As on the original surface of the NNFB; however, a characteristic peak of As existed in the region of binding energy 43.2–47.5 eV after being immersed in As(III) of NNFB (**Figure 3B**). The changes in As3d fine spectra indicated that NNFB adsorbed As on its surface. In addition, the Fe2p spectra showed that Fe^3+^ had two characteristic peaks and one satellite peak, among which the peaks of Fe^3+^(2p1/2) and Fe^3+^(2p3/2) were 711.36 and 724.79 eV, respectively, and the satellite peak of Fe^3+^ was 718.99 eV (**Figure 3C**). After adsorption of As, the peak of Fe2p energy spectrum of the NNFB migrated to the low field, which showed that the peaks of Fe^3+^(2p1/2) and Fe^3+^(2p3/2) were 711.17 and 724.3 eV, respectively, and the satellite peak of Fe^3+^ was 714.16 eV (**Figure 3C**). The results indicated that the electron density surrounding the iron atom in NNFB significantly increased following As adsorption. Thus, we hypothesized that the γ-Fe_2_O_3_ in NNFB might play crucial roles in the process of As adsorption, which is consistent with previous studies indicating that iron oxides and their hydroxides possess excellent adsorption capacities for As. Collectively, the NNFB had the ability to adsorb As and the pore structure on the surface NNFB was reduced after adsorbed As, which might play a role in the treatment of industrial wastewater and As-contaminated farmland.

### 3.3 The NNFB promotes seedling development of rice

**Figure 4:**
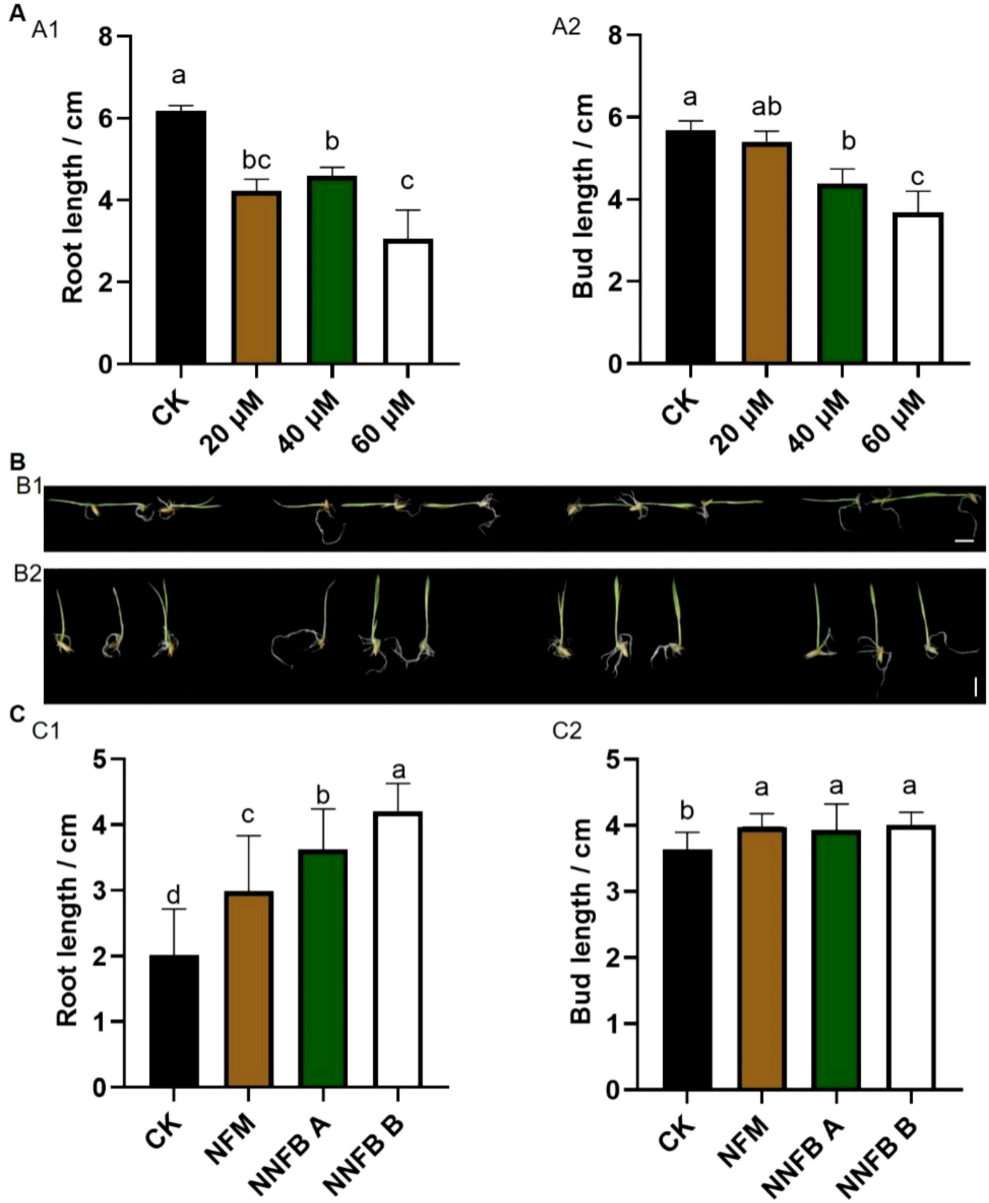
Comparison of seedling growth after treatment with NNFB under arsenic stress at seed germination stage in rice. **(A)** Comparison of the root (A1) and bud length (A2) of rice after 7 days of germination under treatment with 0, 20, 40, and 60 μM As(III), respectively (n=8). Duncan’s test, *p* < 0.05. **(B)** Phenotypes of rice after 7 days of germination under treatment with 40 μM As(III) alone (CK) and the combination of 40 μM As(III) with either 0.15% NNFB, NNFB A, or NNFB B. Bar = 1 cm. **(C)** Comparison of the root (C1) and bud length (C2) of rice after 7 days of germination under treatment with 40 μM As(III) alone (CK) and the combination of 40 μM As(III) with either 0.15% NNFB, NNFB A, or NNFB B, respectively (n=14). Duncan’s test, *p* < 0.05.

To investigate the impact of NNFB at rice seed germination stage, 0% of NNFB (the control groups, CK) and the groups treated with 0.15% nano-ferro-silicon material (NFM), 0.15% of NNFB A (containing 14.28% mass fraction of biomass), and 0.15% of NNFB B (containing 50% mass fraction of biomass) were carried out in rice seed germination. The results showed that 0.15% NFM had no obvious negative effect and even promoted the growth of seed buds and roots at germination stage. The 0.15% NNFB A and NNFB B promoted bud growth while having no effect on root development (**Supplementary Figure S1**). In addition, the bud and root length of 7-day-old seedings were inhibited to varying degrees when applying As(III) at different concentrations of 20, 40, and 60 μM (**Figure 4A**).

To investigate the influence of NNFB at rice seed germination stage under As(III) stress, 0% of NNFB (CK), 0.15% of NFM, 0.15% of NNFB A, and 0.15% of NNFB B were added into the nutrient solution with 40 μM As(III). The results demonstrated that root length was increased extensively after treatment by 0.15% NFB, NNFB A, and NNFB B compared to CK (**Figures 4B1, C1**), while the bud length increased significantly compared to CK in the treatments with 0.15% NFB, NNFB A, and NNFB B (**Figures 4B2, C2**). These observations indicated that all three materials (NFB, NNFB A, and NNFB B) exhibited the potential to alleviate arsenic stress and promoted the growth of seed buds and roots at germination stage. Notably, NNFB B showed the highest efficacy in alleviating As stress to rice at the seed germination stage.

### 3.4 The NNFB alleviates the effect of As stress and regulates ROS balance in rice under arsenic stress at seedling stage

**Figure 5:**
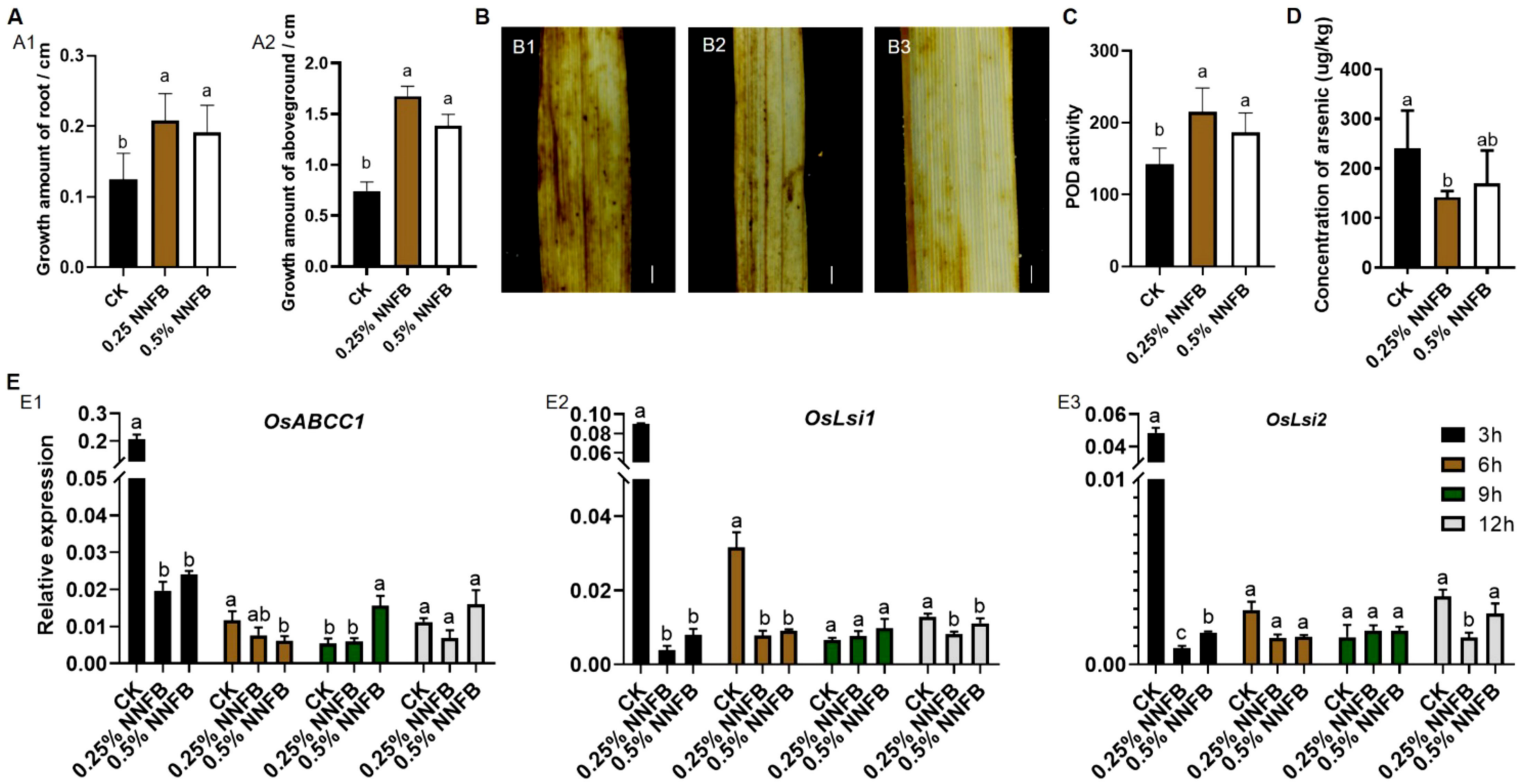
Comparison of the growth of 21-day-old seedlings and the expression level of As transport genes in rice after treatment with NNFB under arsenic stress. **(A)** Comparison of the root (A1) and aboveground length (A2) of 21-day-old seedlings after treatment with 40 μM As(III) alone (CK) and the combination of 40 μM As(III) with 0.25% or 0.5% NNFB for 3 days (n=12). Duncan’s test, *p* < 0.05. **(B)** DAB staining of leaves from 21-day-old seedlings treated with 40 μM As(III) alone (CK) and the combination of 40 μM As(III) with 0.25% or 0.5% NNFB for 3 days. (B1) 40 μM As(III); (B2) 40 μM As(III) + 0.25% NNFB; (B3) 40 μM As(III) + 0.5% NNFB. Bar = 1 mm. **(C)** Comparison of POD enzyme activity in leaves of 21-day-old seedlings after 3 days treatment with 40 μM As(III) alone and the combination of 40 μM As(III) with 0.25% or 0.5% NNFB, respectively. **(D)** Comparison of total As content in rice seedlings after 3 days treatment with 40 μM As(III) alone and the combination of 40 μM As(III) with 0.25% or 0.5% NNFB, respectively. Duncan’s test, *p* < 0.05. **(E)** Comparison of the relative expression level of *OsABCC1* (E1), *OsLsi1* (E2), and *OsLsi2* (E3) in roots of 21-day-old rice seedlings after different time points (3, 6, 9, and 12 h) under treatment with 40 μM As(III) alone and the combination of 40 μM As(III) with 0.25% or 0.5% NNFB, respectively.

At the seedling stage, rice exhibited enhanced vitality and stress resistance compared to that at germination stage. Therefore, concentrations of 0.25% and 0.5% NNFB were selected to evaluate their effects on growth and development of 21-day-old rice seedlings. The results showed that the growth rates of both root and aboveground were significantly increased after the treatments with 0.25% and 0.5% NNFB compared to CK (**Supplementary Figure S2**). Under treatment with 40 μM As(III), the growth amount of root and aboveground were increased significantly when added with 0.25% and 0.5% NNFB (**Figure 5**). In addition, the mean increment of root and aboveground were 4.25 and 1.27, and 3.75 and 1.86 times higher when applying 0.25% and 0.5% of the NNFB, respectively, compared to that in CK (**Figure 5A**). The results indicated that the supplementation of 0.25% and 0.5% NNFB into rice nutrient solution mitigated the inhibitory effects of As stress on growth of both roots and aboveground parts in rice seedlings.

The toxicity of heavy metals primarily manifests through the induction of reactive oxygen species (ROS), such as hydrogen peroxide (H_2_O_2_) (Gadjev et al., 2008). Peroxidase (POD) and catalase (CAT) are key antioxidative enzymes that regulate H_2_O_2_ metabolism in plants (Shao et al., 2007). DAB staining revealed that the control group, consisting of 21-day-old seedlings treated with 40 μM As(III), exhibited the deepest dyeing intensity in their leaves. However, the staining intensity was extensively reduced when 0.25% and 0.5% NNFB were added, suggesting a decrease in H_2_O_2_ content in the leaves (**Figure 5B**). In addition, the POD activity was significantly increased following the addition of two concentrations of NNFB, in comparison to the control group (**Figure 5C**), while no difference in CAT activity was observed (**Supplementary Figure S3**). Generally, the NNFB alleviated As toxicity in rice seedlings by reducing ROS and increasing POD activity. Moreover, the total As content in rice seedlings was decreased when added 0.25% and 0.5% of NNFB in 40 μM As(III) treatment (**Figure 5D**). Consistently, the transcript levels of As transport genes *OsLsi1*, *OsLsi2* (Ma et al., 2008), and *OsABCC1* (Song et al., 2014) were markedly downregulated in 40 μM As(III) + 0.25% or 0.5% of NNFB treatments (**Figure 5E**), and the expression of *OsABCC1* was significantly higher after treatment with 40 μM As(III) + 0.5% of NNFB for 9 h (**Figure 5E1**), suggesting that the reduction in As accumulation following NNFB supplementation was attributed to the altered expression of As transport genes in rice under As stress.

### 3.5 The NNFB increases rice yield in arsenic-contaminated soil at maturity stage

**Figure 6:**
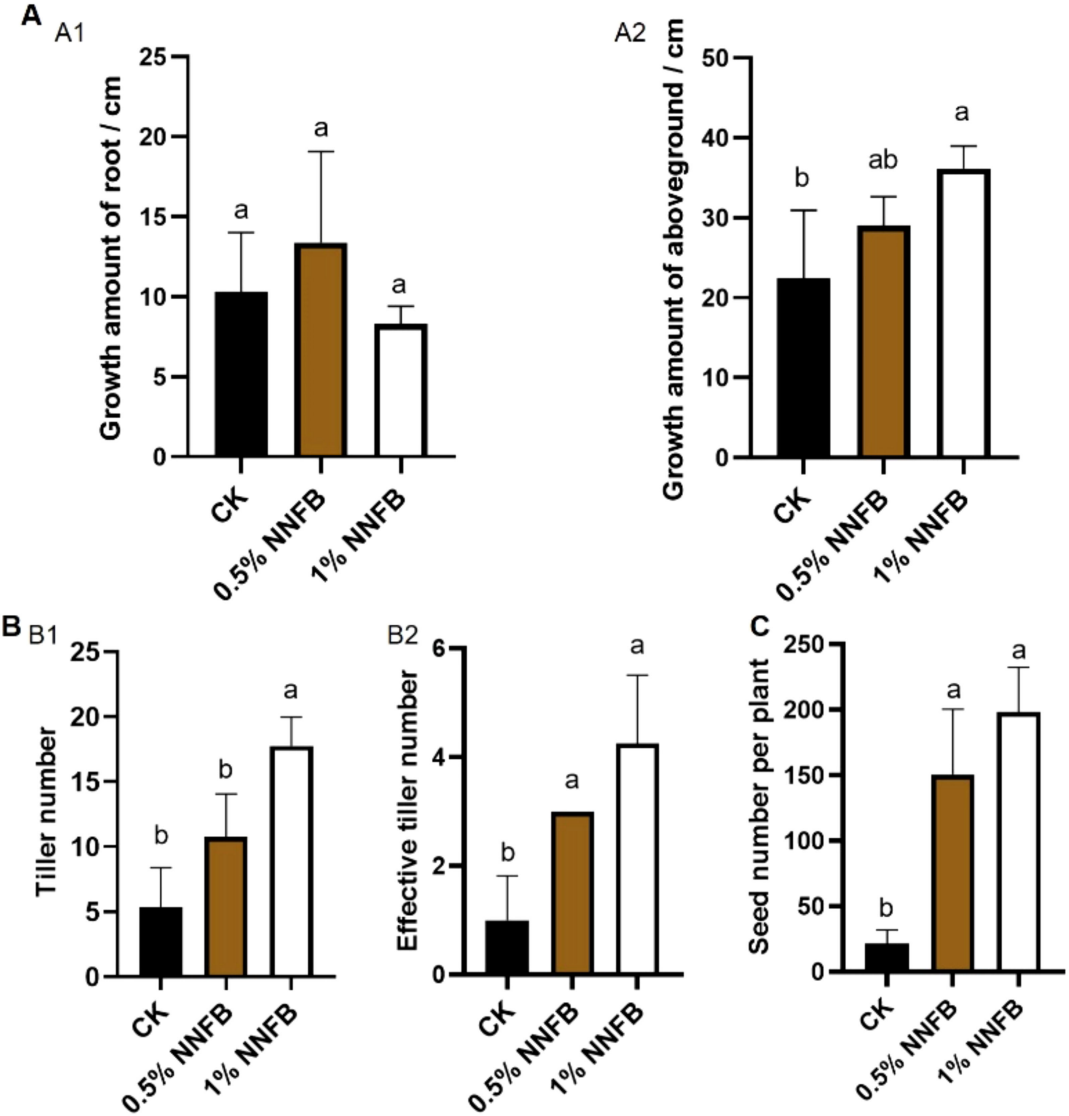
Comparison of the growth and yield traits of rice plants grown in arsenic contaminated soil with or without addition of NNFB. **(A)** Comparison of the growth amount of root (A1) and aboveground (A2) of rice plants grown in soils treated with 40 mg/kg As(III) without or with 0.5% or 1% NNFB. **(B)** Comparison of tiller number (B1) and effective tiller number (B2) of rice plants grown in soils treated with 40 mg/kg As(III) without or with 0.5% or 1% NNFB. **(C)** Comparison of seed number per plant of rice plants grown in soils treated with 40 mg/kg As(III) without or with 0.5% or 1% NNFB.

To explore the effect of NNFB on rice yield in arsenic-contaminated soil, the plants were cultivated in soil containing 40 mg/kg As(III) along with three distinct concentrations of NNFB [0% (control group, CK), 0.5%, and 1%]. Subsequently, a phenotypic comparison of mature rice plants was examined under varying growth conditions in pot experiment. Although no significant difference was observed in root growth after the application of 0.5% and 1% of NNFB compared to CK (**Figure 6A1**), the aboveground biomass in 1% NNFB (36.16 ± 2.80 cm) was significantly increased than that in CK (22.38 ± 8.57 cm) (**Figure 6A2**). In addition, the application of 1% NNFB led to a significant increase in both total tillering number and effective tillering number compared to CK (**Figure 6B**). The effective tillering number remained consistent between the two applied concentrations (0.5% and 1%) of NNFB in soil containing 40 mg/kg As(III) (**Figure 6B2**). Importantly, the seed number per plant was 6.93 and 7.93 times more in 0.5% and 1% NNFB (150.75 ± 49.84 and 172.5 ± 58.65, respectively) than that in CK (21.75 ± 10.24) (**Figure 6C**). The increase in tillering number, effective tillering number, and seed number per plant indicated that the application of NNFB to arsenic-contaminated soil can effectively alleviate As stress and increase rice yield.

### 3.6 A working model of NNFB manufactured and promoted development in rice under arsenic stress

**Figure 7:**
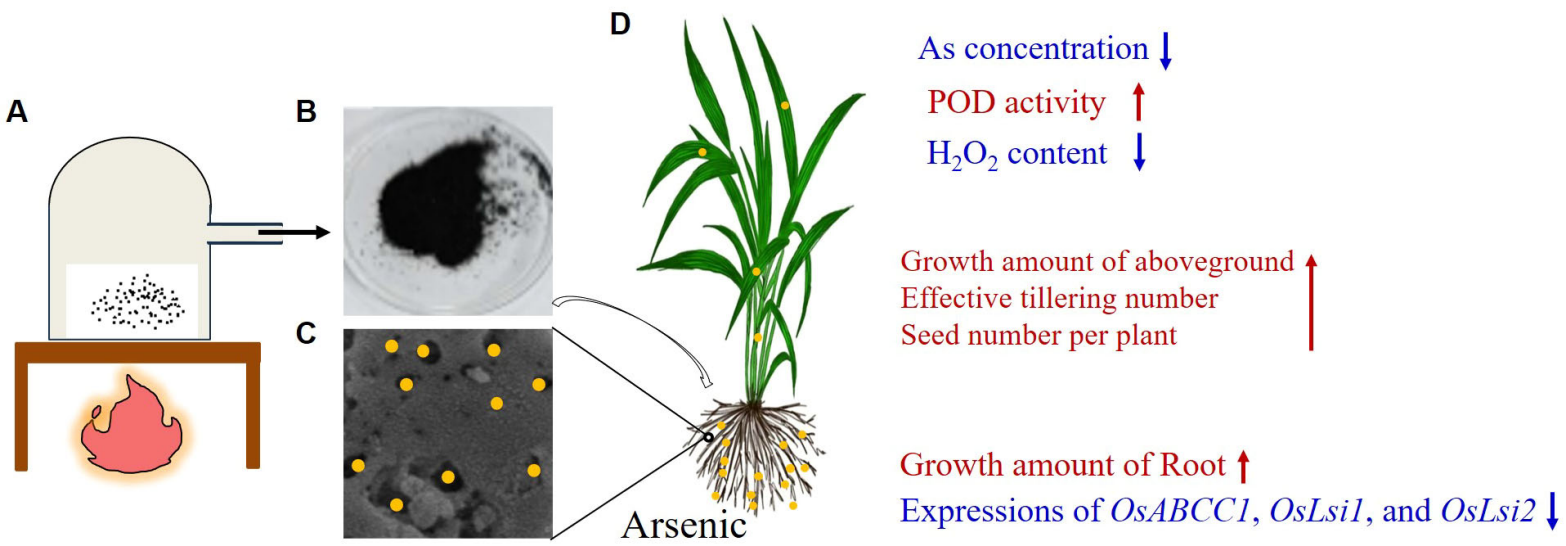
Manufacturing and working model of NNFB. **(A)** Manufacturing model of NNFB. **(B)** Production of NNFB. **(C)** Magnification model of NNFB that adsorbs As(III). **(D)** The rice plant treated with NNFB under As stress. Yellow circles represent the As ionic. The As concentration, H_2_O_2_ content, and the expressions of *OsABCC1*, *OsLsi1*, and *OsLsi2* were decreased, and the POD activity, growth amount of aboveground and root, effective tillering number, and seed number per plant were increased after NNFB treatment. The blue and red fonts mean decrease and increase, respectively.

As described above, we obtained the NNFB that was manufactured by mainly using TEOS, Fe(NO_3_)_3_, and rice straw powder, and fired at 600°C without air for 4 h in a muffle furnace and wrapped in tinfoil tightly (**Figures 1**, **7A**). The NNFB was black particles with obvious pore structure in the irregularity surface, which increased the specific surface area, to adsorb As(III) (**Figures 7B, C**). The NNFB alleviated the effect of As toxicity at rice seed germination and seedling stages. When the NNFB was added into the environment containing 40 μM As(III), the ROS homeostasis was regulated by reducing H_2_O_2_ content and increasing POD activity, which in turn caused the root and aboveground growth to be repaired compared with the rice seedling in 40 μM As(III) (**Figures 6A1**, **7D**), and the As concentration was reduced via regulating expression of the As transport genes (*OsABCC1*, *OsLsi1*, and *OsLsi2*) at rice seedling stage (**Figures 6A2, B**, **7D**). The tiller number, effective tiller number, and seed number per plant were increased when NNFB was added in arsenic-contaminated soil (**Figure 7D**). Collectively, the NNFB manufactured here could adsorb As, promote the plant development at different stages, and increase yield in rice.

## 4 Discussion

### 4.1 The NNFB possesses the ability to adsorb As(III) and promote rice growth

Biochar is an effective material for soil bioremediation, carbon sequestration, phytoremediation, and industrial wastewater treatment, and has received extensive attention in recent years (Tang et al., 2013; Zhang et al., 2023; Fan et al., 2024; Zhao et al., 2025). Due to its high specific surface area with abundant pore structure and functional groups, the biochar exhibits exceptional adsorption capacity for heavy metal cations and is widely utilized for the remediation of water and soil contaminated by pollutants (Lehmann et al., 2006; Zhang et al., 2023). In this study, the TEOS, Fe(NO_3_)_3_·9H_2_O, and the rice straw powder after grinding were used as main raw materials to manufacture NNFB, which contains γ-Fe_2_O_3_ and SiO_2_ by XRD and XPS analysis (**Figures 2B, C**). Because it contained some nutrient elements and inorganics, the bud lengths **(Supplementary Figure S1**) and the growth amounts of root and aboveground were increased than in CK after the supplementation of 0.25% and 0.5% of NNFB at the seedling stage of rice (**Supplementary Figure S2**), indicating that the NNFB could promote rice growth.

### 4.2 The NNFB alleviates the toxicity of arsenic at different development stages of rice

Biochar is abundant in organic matter and inorganic salts, making it a valuable fertilizer that can be readily assimilated by plants and microorganisms (Duwiejuah et al., 2020; Ding et al., 2016; Zhang et al., 2025). In this study, the addition of NNFB to the nutrient solution containing As(III) mitigated the adverse effects of arsenic contamination on rice plant growth and development, as evidenced by improved performance during both the seed germination and seedling stages (**Figures 4**, **5B**) and extensively reduced As absorption and accumulation in rice seedlings (**Figures 5D, E**). In addition, the expressions of As(III) uptake and transport genes *OsLsi1*, *OsLsi2*, and *OsABCC1* were downregulated in rice roots cultivated in As-contaminated nutrient solution after treatment with different concentrations of NNFB. Moreover, a relatively high concentration of H_2_O_2_ was found to have accumulated in the leaves of rice seedlings that were cultured in an environment of 40 μM As(III), while the level of H_2_O_2_ was decreased and the POD activity was increased after the addition of 0.25% and 0.5% of NNFB (**Figures 5B, C**). The previous study on the application of FeO nanomaterials to rice seedlings under As stress obtained the similar results, showing increased ROS scavenging enzyme activity and enhanced seedling vitality (Chatterjee et al., 2021). Those findings suggest that NNFB mitigates As stress by regulating expression of genes involved in regulating As uptake, translocation, and accumulation in rice. Importantly, compared with a zinc oxide nanomaterial (Lee et al., 2010), the NNFB does not produce obvious nanomaterial toxicity in rice while alleviating the arsenic stress of rice seedling. Thus, the application of NNFB promotes rice growth and alleviates the effect by As pollution at different developmental stages in rice, making it more effective for rice production.

In the practice of grain production, plant growth would be stunted or delayed under As stress, which resulted in a variety of metabolic pathways inhibited, and reduced production or even no fruit, eventually. The application of 0.5% and 1% of NNFB increased tiller number, effective tiller number, and seed number per plant of rice in arsenic-contaminated soil (**Figures 6B, C**), indicating that NNFB alleviates the toxic effect and increases the gain yield in rice under As stress.

## 5 Conclusion

We designed and synthesized NNFB with γ-Fe_2_O_3_ and SiO_2_ as main components. Because of adsorption to As, NNFB has certain application potential in treating industrial wastewater and As-contaminated farmland. In plant, the NNFB alleviates As stress at seed germination, seedling, and mature stages of rice under arsenic-contaminated environment and regulates the ROS balance and prevents As absorption by downregulating the expression of genes involved in As uptake, translocation, and accumulation in rice under As stress. In addition, both 0.5% and 1% NNFB could increase the growth amount of aboveground, the effective tillering number, and the seed number per plant in the soil contained 40 mg/kg As(III). The facilitating effect of 1% NNFB was more significantly than 0.5%. In a word, the NNFB that we manufactured in this study has the ability to alleviate As stress and promote the plant growth and grain production of rice; thus, it has an application value for increasing rice production and ensuring food security.

## Data Availability

The original contributions presented in the study are included in the article/**Supplementary Material**. Further inquiries can be directed to the corresponding author.
