# Supplementary material for: New nano-ferro-silicon biochar promotes plant growth and grain yield under arsenic stress in rice
Source: Front Plant Sci. 2025 May 2;16:1556696. doi: 10.3389/fpls.2025.1556696 (PMC12081388; doi:10.3389/fpls.2025.1556696)
Supplement: Supplementary file 1 [file DataSheet1.pdf]

Supplementary Materials

New nano-ferro-silicon biochar promotes plant growth and grain yield under arsenic stress in rice

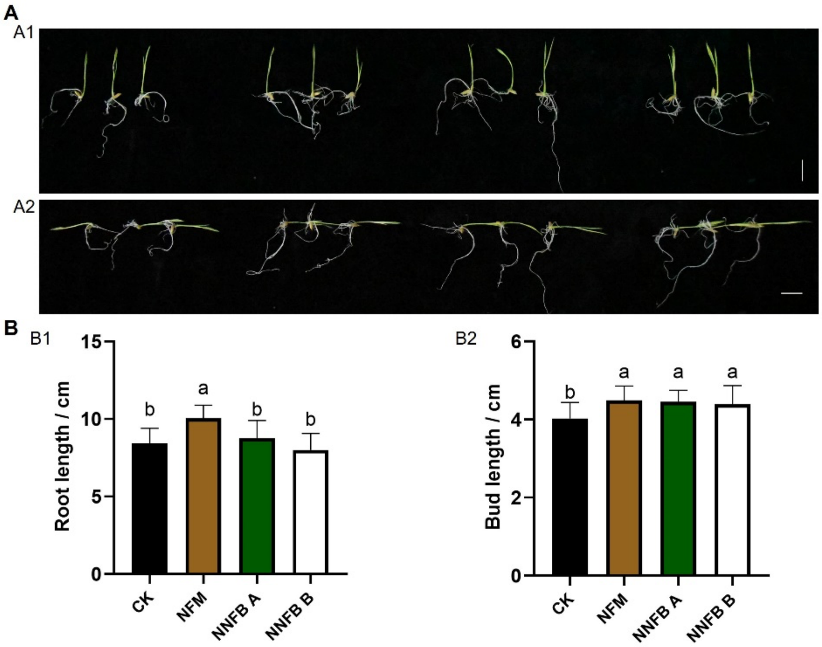

Figure S1. The NNFB promotes rice seedling growth at the seed germination stage.

A, Phenotypes of rice after 7 days of germination treated with 0.15% of NNFB, NNFB A, NNFB B. Bar = 1 cm. **B**, Comparison of root (B1) and bud length (B2) after treatment with 0.15% of NNFB, NNFB A, NNFB B (n=14). Duncan's test, $p < 0.05$.

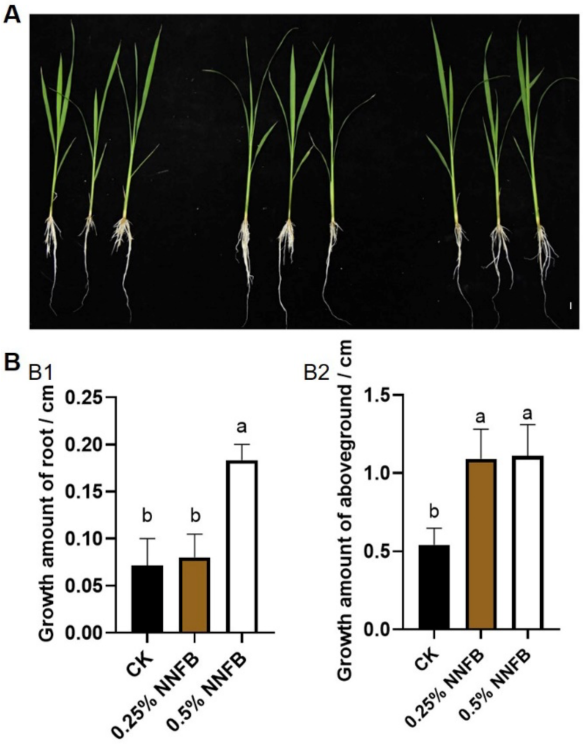

Figure S2. **The NNFB promotes the growth of 21-day-old seedlings in rice.**

A, Phenotypes of 21-day-old rice seedlings treated with 0.25% and 0.5% of NNFB for 3 days. From left to right are 0% of NNFB (CK), 0.25% of NNFB, and 0.5% of NNFB. Bar = 1 cm. **B**, Comparison of the growth amount of root (B1) and aboveground (B2) of 21-day-old rice seedlings after 3 days treatment with 40 μM As(III) alone (CK) and the combination of 40 μM As(III) and either 0.25% or 0.5% of NNFB, respectively (n=12). Duncan's test, $p < 0.05$.

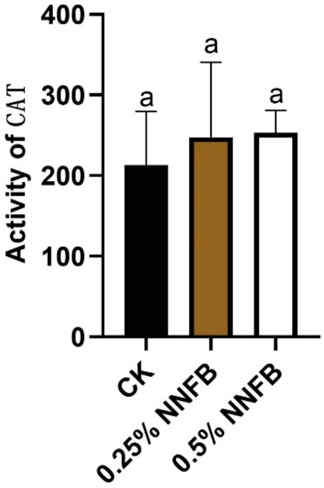

Figure S3. Comparison of the CAT enzymic activity in leaves of 21-day-old rice seedlings after 3 days treatment with 40 μM As(III) without or with 0.25% and 0.5% of NNFB.

Table S1. **The composition of nutrient solution used in rice hydroponic cultivation**

Reagent	g / 10 L
NH_4NO_3	914
$NaH_2PO_4·2H_2O$	403
K_2SO_4	714
$CaCl_2$	886
$MgSO_4·7H_2O$	3240
$MnCl·4H_2O$	15
$(NH_4)_6Mo_7O_{24}·4H_2O$	0.74
H_3BO_3	9.34
$ZnSO_4·7H_2O$	0.35
$CuSO_4·5H_2O$	0.31
$FeSO_4·7H_2O$	69.5
EDTA-2Na	93.06

Table S2. **Treatments of rice seedlings by different concentration of NNFB**

Group	Treatment
CK	1.6 L rice nutrient solution
Group 1	1.6 L rice nutrient solution + 0.25% (4 g) NNFB contained 50% biomass
Group 2	1.6 L rice nutrient solution + 0.5% (8 g) NNFB contained 50% biomass
Group 3	1.6 L rice nutrient solution + 40 μM $AsNaO_2$
Group 4	1.6 L rice nutrient solution + 40 μM $AsNaO_2$ + 0.25% (4 g) NNFB contained 50% biomass
Group 5	1.6 L rice nutrient solution + 40 μM $AsNaO_2$ + 0.5% (8 g) NNFB contained 50% biomass

Table S3. **Primers used in this study**

Primer	Sequence (5'-3')	Function
OsABCC1-F	AACAGTGGCTTATGTTCCTCAAG	qRT-PCR
OsABCC1-R	AACTCCTCTTTCTCCAATCTCTG	qRT-PCR
OsLsi1-F	GATGTCGTCGATCGTGTCCTCTTAC	qRT-PCR
OsLsi-R	CACACAAAGACGTAGCTAGTGATG	qRT-PCR
OsLsi2-F	CCTTGCTTGCATGCATGCATGGTG	qRT-PCR
OsLsi2-R	CTAGCTAGCTAGCTCCATCTAGTTC	qRT-PCR
OsUBQ-F	CACCCTGGCTGACTACAACA	qRT-PCR
OsUBQ-R	TTCTTCTTGCGGCAGTTGAC	qRT-PCR
